# High Pressure Behavior of Chromium and Yttrium Molybdate (Cr_2_Mo_3_O_12_, Y_2_Mo_3_O_12_)

**DOI:** 10.3389/fchem.2018.00478

**Published:** 2018-10-11

**Authors:** Lindsay Young, Jennifer Gadient, Cora Lind

**Affiliations:** Department of Chemistry and Biochemistry, The University of Toledo, Toledo, OH, United States

**Keywords:** negative thermal expansion, high pressure, synchrotron radiation, *in-situ* studies, scandium tungstate family

## Abstract

The high pressure behavior of negative thermal expansion materials continues to be of interest, as their potential use in controlled thermal expansion composites can be affected by irreversible pressure-induced phase transitions. To date, it is not possible to predict the high pressure behavior of these compounds, necessitating measurements on each composition. In this work, high pressure synchrotron powder X-ray diffraction studies of Cr_2_Mo_3_O_12_ and Y_2_Mo_3_O_12_ were conducted in a diamond anvil cell. Chromium molybdate, which adopts the monoclinic P2_1_/a structure under ambient conditions, was found to not undergo any crystalline-crystalline transitions up to 8.9 GPa. The orthorhombic ambient pressure polymorph of yttrium molybdate was found to undergo a phase transition to the monoclinic P2_1_/a scandium tungstate structure below 0.13 GPa. This structure is frequently observed for related materials at low temperatures, but has never been reported for Y_2_Mo_3_O_12_. No additional changes in this material were observed up to 4.9 GPa. The fact that the monoclinic polymorphs of these materials do not undergo phase transitions within the studied pressure range makes them unique among A_2_M_3_O_12_ materials, as most isostructural compositions undergo at least one phase transition to crystalline high pressure phases.

## Introduction

Thermal expansion describes the tendency of materials to change dimensions with increasing temperature. Due to longitudinal vibrations of atoms along atomic bonds as thermal energy is introduced, most materials expand. The thermal expansion coefficient α quantifies the magnitude of dimensional change over a specific temperature range. Mismatches in thermal expansion are a major concern in many engineering fields for any devices that combine two or more materials (Roy et al., 1989; Lommens et al., 2005; Takenaka, 2012). The differences in the magnitude of thermal expansion between two adhered materials can lead to degradation of devices with thermal cycling due to delamination at the interface. In addition, any optical or electronic applications where absolute dimensions are crucial for optimal performance require materials that display negligible expansion to ensure dimensional stability. These challenges have led to significant interest in materials that display negative thermal expansion (NTE) (Korthuis et al., 1995; Evans et al., 1996; Attfield and Sleight, 1998a,b; Lind et al., 1998, 2011; Sleight, 1998; Reisner et al., 2000; Li et al., 2002; Phillips et al., 2008; Chapman and Chupas, 2009; Kozy et al., 2009; Greve et al., 2010). It has been proposed that NTE materials when incorporated as fillers in composites would allow the overall expansion of the material to be reduced or tailored to a specific value (Verdon and Dunand, 1997; Holzer and Dunand, 1999; Matsumoto et al., 2003; Sullivan and Lukehart, 2005; Tani et al., 2007, 2010; Lind et al., 2011). This has been accomplished previously with zirconium tungstate as a filler in a ceramic zirconia optical fiber coating (Fleming et al., 1997). However, other attempts to prepare composites with tailored expansion coefficients have failed due to irreversible phase transitions of the NTE filler under the temperature and pressure conditions encountered during manufacturing or use. For example, a Cu/ZrW_2_O_8_ composite showed highly irreproducible expansion behavior due to formation of the orthorhombic high pressure phase of ZrW_2_O_8_, which displays positive volume expansion (Holzer and Dunand, 1999).

There are several classes of materials that display NTE properties. One of these is the scandium tungstate family. These materials are often referred to as the A_2_M_3_O_12_ family, which includes a wide range of compositions, as A can be any trivalent cation ranging in size from Al^3+^ to the smaller lanthanides, and M can be molybdenum or tungsten. These materials crystallize in corner-sharing networks of AO_6_ octahedra and MO_4_ tetrahedra, and many compositions form closely related monoclinic (P2_1_/a-A_2_M_3_O_12_) and orthorhombic (Pbcn-A_2_M_3_O_12_) structures. NTE is only observed in the orthorhombic phase, and arises from concerted tilting motions of the polyhedra. The formation of the monoclinic and orthorhombic phases depends heavily on composition, and many compounds show a reversible transition between the monoclinic polymorph at low temperatures and the orthorhombic structure at high temperatures. The temperature at which this transition occurs varies widely with composition, and in extreme cases, materials can adopt the monoclinic or orthorhombic structures over their entire stability range. For instance, scandium tungstate, yttrium tungstate and yttrium molybdate (Nassau et al., 1971; Evans et al., 1998; Forster and Sleight, 1999; Marinkovic et al., 2005; Zhou et al., 2008) retain the orthorhombic structure to at least −263 to −258 °C, the lowest temperatures studied to date, while gallium molybdate remains monoclinic up to its decomposition temperature of 600 °C (Gates et al., 2006).

In addition to the corner-sharing orthorhombic and monoclinic polymorphs described above, denser structures with higher A-site coordination numbers, resulting in a combination of both corner- and edge-shared polyhedra, are known for compositions that contain the larger lanthanides lanthanum through terbium (Nassau et al., 1965, 1971). Yttrium's ionic radius falls between the ionic radii of the trivalent lanthanides that form the Pbcn structure and polymorphs with 7- or 8-coordinated A^3+^ cations, respectively (Shannon, 1976). As a result, yttrium molybdate can adopt two orthorhombic structures in space groups Pba2 and Pbcn under ambient conditions (Marinkovic et al., 2005; Gates and Lind, 2007). Pbcn-Y_2_Mo_3_O_12_ is thermodynamically stable above 550 °C but can be retained as a metastable phase by quenching to room temperature (Gates and Lind, 2007). This structure readily absorbs water from the atmosphere, leading to formation of a trihydrate, Y_2_Mo_3_O_12_·3H_2_O (Kol'tsova, 2001). The denser Pba2-phase is isostructural to Tb_2_Mo_3_O_12_, with a higher coordination number for Y and edge sharing YO_7_ polyhedra. The denser Pba2-Y_2_Mo_3_O_12_ polymorph is thermodynamically stable below 550 °C, but is kinetically disfavored, thus requiring long periods of annealing at 530 °C to prepare it. This structure does not hydrate (Gates and Lind, 2007).

Because NTE materials may need to withstand high pressures and temperatures during production and regular use of composites, it is important to characterize their behavior under non-ambient conditions for effective application. The open framework structure of these materials that gives rise to NTE is highly susceptible to pressure-induced changes. High pressure studies of a number of NTE materials have shown that they undergo phase transitions to high pressure polymorphs or amorphize (see Table 1 and references therein). These denser structures are not expected to exhibit NTE (Hu et al., 1997; Paraguassu et al., 2004; Garg et al., 2005b; Maczka et al., 2012), as the phonon modes that cause NTE require corner-sharing open frameworks. Irreversible phase transitions to high pressure polymorphs are thus detrimental for potential applications, while reversible phase transitions may be acceptable if they occur at pressures that are not encountered during use of composites.

**Table 1:** Current high pressure research on scandium tungstate family materials.

**Material**	**Method**	**PTF**	**Transition (GPa)**	**Study findings/Cell**	**R^†^**
AlFeMo_3_O_12_	PXRD (Young et al., 2016)	M:E	1.7	α' transition from isotropic to anisotropic compression	R
			3.2	P2_1_/a γ-phase a = 14.94 Å, b = 8.71 Å, c = 17.48 Å, β = 124.4°	R
			3.9-4.2	Monoclinic δ-phase, a = 14.20 Å, b = 8.36 Å, c = 13.57 Å, β = 102.0°	R, H
AlGaMo_3_O_12_	PXRD (Young et al., 2016)	M:E	2.0, 2.9	α' and α” transition from isotropic to anisotropic compression	R
			4.1–4.4	P2_1_/a γ-phase a = 14.76 Å, b = 8.59 Å, c = 17.21 Å, β = 124.6°	R
			5.0–5.3	Monoclinic δ-phase a = 14.16 Å, b = 8.32 Å, c = 13.62 Å, β = 102.2°	R, H
Al_2_Mo_3_O_12_	PXRD (Young et al., 2016)	M:E	3.0	α' transition from isotropic to anisotropic compression	R
			4.6–4.9	P2_1_/a γ-phase a = 14.65 Å, b = 8.53 Å, c = 17.08 Å, β = 124.5°	R
			5.7–6.1	Monoclinic δ phase a = 14.04 Å, b = 8.28 Å, c = 13.47 Å, β = 101.9°	R, H
Al_2_W_3_O_12_	Raman (Garg et al., 2001)	M:E	0.05	Unindexed phase	R < 2
			7	Amorphization	I
	PXRD (Achary et al., 2002)	None	8, 48 h	ambient phase recovered	
	PXRD/900°C (Achary et al., 2002)	None	3	Decomposes to AlWO_4_ + WO_3−x_	
	AC resistivity (Mukherjee et al., 2003)	None	0.5	Phase transition	I
	Raman (Maczka et al., 2004)	M:E	0.28	Potentially monoclinic	R
			2.8	Unindexed	
	PXRD/ dielectric measurement (Mukherjee et al., 2004)	M:E	0.5	P 2_1_ a = 8.95 Å, b = 9.07 Å, c = 12.59 Å, β = 90.51°	ND
			3.4	P 2_1_ a = 9.59 Å, b = 12.52 Å, c = 7.84 Å, β = 91.99°	ND
			6–18	Amorphization	I
	Raman (Garg et al., 2005b)	M:E	0.4–3	Unindexed phase	R
			5.3–6	Unindexed phase	ND
			14	Amorphization	I
	PXRD (Varga et al., 2005a)	iPrOH	0.1	P2_1_/a a = 15.41 Å, b = 9.05 Å, c = 19.91 Å, β = 125.4°	R
			7	Amorphization	I
Fe_2_Mo_3_O_12_	PXRD (Young et al., 2016)	M:E	1.5	α' transition from isotropic to anisotropic compression	R
			2.7–2.9	P2_1_/a γ-phase a = 15.06 Å, b = 8.79 Å, c = 17.63 Å, β = 124.6°	R
			3.5–3.7	Monoclinic δ phase a = 14.46 Å, b = 8.48 Å, c = 13.77 Å, β = 102.0°	R, H
	Raman (Moura et al., 2016)	Mineral oil	4.8	Amorpization	R
Ga_2_Mo_3_O_12_	PXRD (Gates et al., 2006)	M:E	3.2	Monoclinic phase a = 13.7 Å, b = 7.3 Å, c = 12.3 Å, β = 115.9°	R
			4.2	a = 14.4 Å, b = 8.3 Å, c = 13.8 Å, β = 103.1°	R
			8	Amorphization	I
	PXRD (Young et al., 2016)	M:E	<3.3	P2_1_/a γ-phase a = 15.24 Å, b = 8.68 Å, c = 17.44 Å, β = 126.1°	
			<4.2	Monoclinic δ-phase a = 14.45 Å, b = 8.34 Å, c = 13.71 Å, β = 101.3°	
In_2_Mo_3_O_12_	Raman (Mendonça et al., 2016)	M:E	1.5	Denser structure	ND
			5–>7	Gradual amorphization	I
In_1.5_Y_0.5_Mo_3_O_12_	Raman (Mendonça et al., 2016)	M:E	1.0	Denser structure	ND
			3.4–5	Two stage amorphization	ND
In_2_W_3_O_12_	PXRD (Baiz, 2010; Baiz et al., 2012)	M:E	1.9–2.7	a = 19.68 Å, b = 4.49 Å, c = 17.34 Å, b = 99.21°	ND
			>2.7	Progressive amorphization	I
Lu_2_W_3_O_12_	PXRD (Liu et al., 2002)	None	5–8	Progressive amorphization	I
Sc_2_Mo_3_O_12_	PXRD and Raman (Arora et al., 2004)	M:E	4–12	Two stage amorphization; distortions and disordering at 4 GPa, complete at 12 GPa	I
	PXRD and Raman (Paraguassu et al., 2004)	M:E Raman	0.29	Unknown potentially monoclinic phase ($C_{2h}^{5}$)	R
			2.7	Unknown phase	R
			3.7–5.1	Amorphization	R < 5
		16:3:1 M:E:H(PXRD)	4–20	Amorphization	I after compression to 20
	PXRD and Raman (Arora et al., 2005)	M:E	12	Amorphization	I
	PXRD (Varga et al., 2005a)	iPrOH	0.25	P2_1_/a a = 16.51 Å, b = 9.54 Å, c = 18.84 Å, β = 125.35°	ND
			2.5–3.0	Unknown phase	ND
			8	Amorphization	Mostly I
Sc_2_W_3_O_12_	Raman (Garg et al., 2001)	M:E	0.45	Unindexed	R
			7	Amorphous	I
	PXRD (Secco et al., 2001)	None	8	Amorphous	I
	PXRD/400°C (Secco et al., 2002a,b)	None	3.2	Unindexed	ND
			4	Amorphous	I
	PXRD/Raman (Garg et al., 2005a)	M:E	0.6	a = 16.0 Å, b = 9.4 Å, c = 18.6 Å, β = 124.9°	R
			1.6	a = 13.81 Å, b = 9.6 Å, c = 18.26 Å, β = 123.91°	I
			6.5–14	Amorphous	I
	PXRD (Varga et al., 2005b)	iPrOH, N_2_	0.3	a = 16.25 Å, b = 9.58 Å, c = 18.94 Å, β = 125.4°	R
			2.8	Unindexed	I
	ND (Varga et al., 2006)	He	0.25–0.3	a = 16.25 Å, b = 9.58 Å, c = 18.93 Å, β = 125.37°	H
	PXRD (Cetinkol et al., 2008)	M:E	0.3	a = 16.25 Å, b = 9.58 Å, c = 18.93 Å, β = 125.38°	
		M:E	2.7	a = 8.45 Å, b = 11.31 Å, c = 9.15 Å, α = 96.6°	I
Pbcn -Y_2_Mo_3_O_12_	Raman (Torres Dias et al., 2013)	Mineral Oil	0.3	Potentially monoclinic, 0.3 GPa	ND
			2.4	Amorphization, 2.4 GPa	I
Y_2_W_3_O_12_	PXRD, Raman (Karmakar et al., 2004)	M:E	>3	Progressive amorphization	I > 4
Zr_2_W_2_PO_12_	PXRD (Cetinkol et al., 2009)	M:E	1.37	a = 9.30 Å, b = 12.10 Å, c = 9.05 Å, β = 89.60°, P2_1_/n11	R
			>3	a = 9.34 Å, b = 11.40 Å, c = 8.21 Å, β = 97.37° P2_1_/n11	R
			>6.3	a = 11.15 Å, b = 9.38 Å, c = 12.52 Å, α = 88.87°, β = 141.65°, γ = 90.93°	R
			>14	Partial amorphization	I

*M:E, 4:1 Methanol:Ethanol; M:E:H, 16:3:1 Methanol:Ethanol:Water*.

† *In the final column, I, irreversible, R, reversible, H, hysteresis, and ND, not determined*.

While a number of high pressure studies of A_2_M_3_O_12_ materials have been conducted, the knowledge of their high pressure behavior is not comprehensive. Some compositions are well characterized, but many are only partially characterized (e.g., In_2_Mo_3_O_12_, In_1.5_Y_0.5_Mo_3_O_12_, Lu_2_W_3_O_12_, Y_2_Mo_3_O_12_, see Table 1) or have not yet been studied (e.g., many Ln_2_M_3_O_12_, Cr_2_Mo_2_O_12_). Variations in data quality and experimental parameters have also led to conflicting results for the same materials (e.g., Al_2_W_3_O_12_, Sc_2_W_3_O_12_, Table 1). As such, the high pressure behavior of A_2_M_3_O_12_ materials remains unpredictable. One exception to this has been the observation that compositions adopting the orthorhombic structure at room temperature undergo a phase transition to the slightly denser P2_1_/a-structure below 0.5 GPa (Paraguassu et al., 2004; Garg et al., 2005a; Varga et al., 2005a,b, 2006; Cetinkol et al., 2008; Varga, 2011; Lind, 2012; Maczka et al., 2012). Generally, at least one additional phase transition occurs at higher pressures between 1 and 4 GPa. Amorphization is commonly reported, which can be reversible or irreversible and shows onsets as low as 2.3 GPa or higher than 10 GPa (Garg et al., 2001, 2005a,b; Secco et al., 2001, 2002a,b; Liu et al., 2002; Arora et al., 2004, 2005; Karmakar et al., 2004; Mukherjee et al., 2004; Paraguassu et al., 2004; Varga et al., 2005a; Gates et al., 2006; Baiz et al., 2012; Torres Dias et al., 2013). Table 1 summarizes the current literature on high pressure behavior of A_2_Mo_3_O_12_compounds.

This paper reports the high pressure behavior of Cr_2_Mo_3_O_12_ and Pbcn-Y_2_Mo_3_O_12_. Cr_2_Mo_3_O_12_ is monoclinic at room temperature and displays positive expansion with α_l_ = 9.8 × 10^−6°^C^−1^ up to 380 °C, where it undergoes a transition to the orthorhombic Pbcn polymorph. Above this temperature, NTE with α_l_ = −9.4 × 10^−6°^C^−1^ is observed (Tyagi et al., 2002). Y_2_Mo_3_O_12_ adopts the orthorhombic structure at all temperatures, and shows NTE with α_l_ = −9.0 × 10^−6°^C^−1^ from −253 to 177 °C (Marinkovic et al., 2009). No previous high pressure studies on chromium molybdate exist, while yttrium molybdate has been investigated by low resolution diffraction studies by our group and Raman spectroscopy by Torres Dias (Gates, 2008; Torres Dias et al., 2013). Our previous low resolution diffraction data showed no evidence of phase transitions until irreversible amorphization occurred at 2.3 GPa. It was surprising that no transition to the monoclinic polymorph was detected. However, the *in situ* Raman study conducted by Torres Dias et al. reported a phase transition at 0.3 GPa, followed by irreversible amorphization at 2.4 GPa. The Raman data suggested a change in symmetry from orthorhombic to monoclinic. In this work, higher resolution synchrotron diffraction data revealed that Pbcn-Y_2_Mo_3_O_12_ indeed undergoes a transition to the monoclinic P2_1_/a structure at low pressure. The atomic coordinates were extracted by Rietveld analysis. Interestingly, P2_1_/a-Y_2_Mo_3_O_12_ and Cr_2_Mo_3_O_12_ do not undergo any further crystalline-crystalline phase transitions before amorphization occurs. To our knowledge, this is the first report of relatively low density corner sharing polyhedral frameworks that remain stable under pressure.

## Experimental

**Figure 1:**
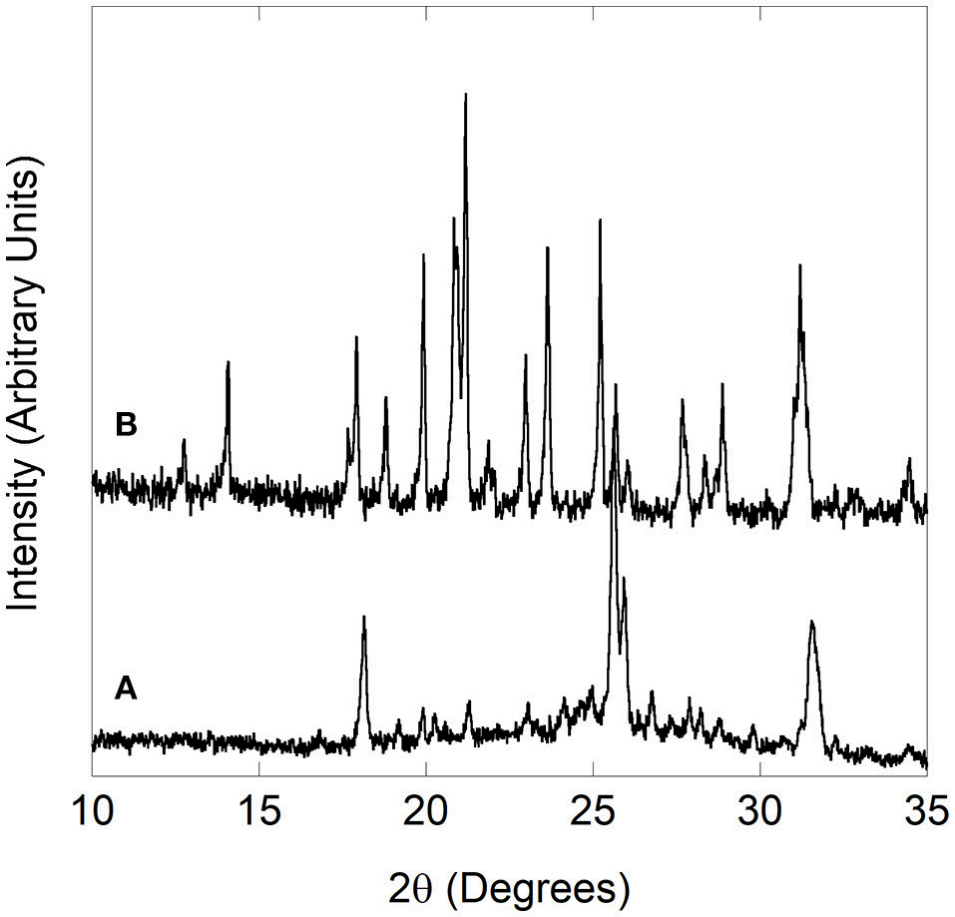
Yttrium molybdate **(A)** trihydrate formed upon exposure to atmospheric moisture, **(B)** Pbcn polymorph reformed after drying.

Cr_2_Mo_3_O_12_ and Pbcn-Y_2_Mo_3_O_12_ powders were synthesized using a non-hydrolytic sol-gel (NHSG) method as described previously (Gates et al., 2006; Gates and Lind, 2007; Gindhart, 2007; Baiz et al., 2008; Gates, 2008; Gindhart et al., 2008; Baiz, 2010). Well crystallized Cr_2_Mo_3_O_12_ was obtained after heat treatment to 500 °C, while Y_2_Mo_3_O_12_ required heating between 800 and 1,000 °C to achieve sharp peaks. Sample quality was confirmed by powder X-ray diffraction on a PANalytical X'Pert Pro Multipurpose Diffractometer. Phase pure samples with good crystallinity were chosen for the high pressure studies. Pbcn-Y_2_Mo_3_O_12_ has a strong tendency to absorb ambient moisture and form a trihydrate (Figure 1A), thus the material was freshly dried (Figure 1B), immediately transferred to a vial while hot and sealed with Parafilm.

*In situ* high pressure powder diffraction studies were carried out at beamline 17-BM at the Advanced Photon Source at Argonne National Laboratory in an EasyLab “Diacell Bragg-(G)” diamond anvil cell (DAC). Data were collected with a 2-D Perkin Elmer a-Si C-window CCD detector during two separate trips. Cr_2_Mo_3_O_12_ was measured at a wavelength of 0.72808 Å and a detector distance of 400 mm. Y_2_Mo_3_O_12_ was investigated during a subsequent trip at a wavelength of 0.72959 Å with a larger detector distance of 600 mm to allow collection of data to smaller d-spacings. Both setups allowed even subtle changes in the PXRD patterns to be seen. Data were collected by averaging six individual 5 s exposures. Anhydrous isopropanol was chosen as a pressure transmitting fluid (PTF) with a hydrostatic limit of 4.2 GPa (Angel et al., 2007). While higher hydrostatic limits can be achieved (Klotz et al., 2009) with alcohol or alcohol/water mixtures (10.5 GPa) or some liquefied inert gases (N_2_: 10 GPa, Ne: 15 GPa, He: 40 GPa), these PTFs are not suitable for the study of many NTE materials. Water is known to penetrate the open frameworks of several NTE compounds, and may lead to formation of crystalline hydrates as observed for ZrW_2_O_8_·H_2_O (Duan et al., 1999; Banek et al., 2010) or Y_2_Mo_3_O_12_·3H_2_O (Kol'tsova, 2001; Marinkovic et al., 2005). While no crystalline methanol adducts have been reported, it is plausible that this molecule may also penetrate into open frameworks and impact the high pressure behavior. Similarly, atomic or diatomic gases may insert into the NTE frameworks, as has been demonstrated for CaZrF_6_ (Hester et al., 2017a).

The powders were finely ground using a mortar and pestle in an approximately 3:1 ratio with sodium chloride as an internal pressure calibrant. An EasyLab “Diacell Bragg-(G)” membrane diamond anvil cell with diamond culet faces measuring 500 microns was fitted to a 300 micron thick steel gasket pre-indented to 100 microns with a 250 micron hole. The sample was packed into the sample chamber, flooded with anhydrous isopropanol as a PTF, and the DAC was quickly sealed. To avoid hydration of Pbcn-Y_2_Mo_3_O_12_, this sample was packed in a glovebag under argon. An “asclosed” pattern was collected before attaching a stainless steel diaphragm to the cell, and a programmable methanol pump was used to gradually increase the pressure to 4.9 GPa (Y_2_Mo_3_O_12_) and 8.9 GPa (Cr_2_Mo_3_O_12_) while collecting data at pressure increments of approximately 0.02 to 0.2 GPa. After the highest pressure was reached, files were collected during decompression in approximately 1.0 GPa steps. Final pressures of 2.2 and 1.9 GPa were observed at the end of the runs for Cr_2_Mo_3_O_12_ and Y_2_Mo_3_O_12_, respectively. A “decompressed” pattern was collected after opening the cell to release any residual pressure. The pressure for each scan was determined from the refined NaCl lattice parameters using the equation of state published by Birch (Birch, 1986). This approach requires calculation of Eulerian strain using equation (1):

(1) $$\begin{array}{l} f\;=\frac{\left({v/v_{o}}^{-2/3}\right)-1}{2} \end{array}$$

where *f* is the Eulerian strain, *v* is the cell volume at pressure P, and *v*_*o*_ is the ambient pressure volume. The value for *v*_*o*_ was determined to be 179.5864 Å^3^. The calculated values for *f* can then be used to determine pressure using equation (2):

(2) $$\begin{array}{l} P={3K_{o}f\left(1+2f\right)}^{5/2}\left(1+af\right) \end{array}$$

where *K*_*o*_ is the bulk modulus at ambient temperature and *a* is a constant that depends on temperature. Literature values for K_o_ (239.9 kbar at 25 °C) and *a* (1.796 at 25 °C) were used (Birch, 1986).

The pressures reported in this manuscript are estimated to have errors of ± 0.1 GPa due to the continuous pressure increase during data collection, which results in each pattern being collected over a small range of pressures. The only exception are scans collected in the “asclosed” cell before attaching the diaphragm, for which we estimate an error of ± 0.02 GPa based on the uncertainty of the extracted lattice parameter of the standard.

Bulk moduli of all phases were estimated using the program PASCal using a 3rd order Birch–Murnaghan equation of state (Cliffe and Goodwin, 2012). For high pressure phases, the first pressure point at which a polymorph was observed was used as critical pressure.

### Data integration and analysis

The 2-D data were integrated using GSAS-II (Toby and Von Dreele, 2013). A pattern of NIST LaB_6_ collected in the DAC was used to calibrate the detector distance and determine the penetration correction, which was necessary to correct a slight non-linear distortion of the data due to penetration of X-rays into the detector. The patterns were refined using Topas Academic (Bruker, 2006; Coelho, 2018), and consecutive refinements were conducted in command line mode by copying each output file to the input file for the next dataset. Rietveld refinements were carried out for all phases, as the atomic coordinates were either known or determined in the course of this work.

## Results and discussion

**Figure 2:**
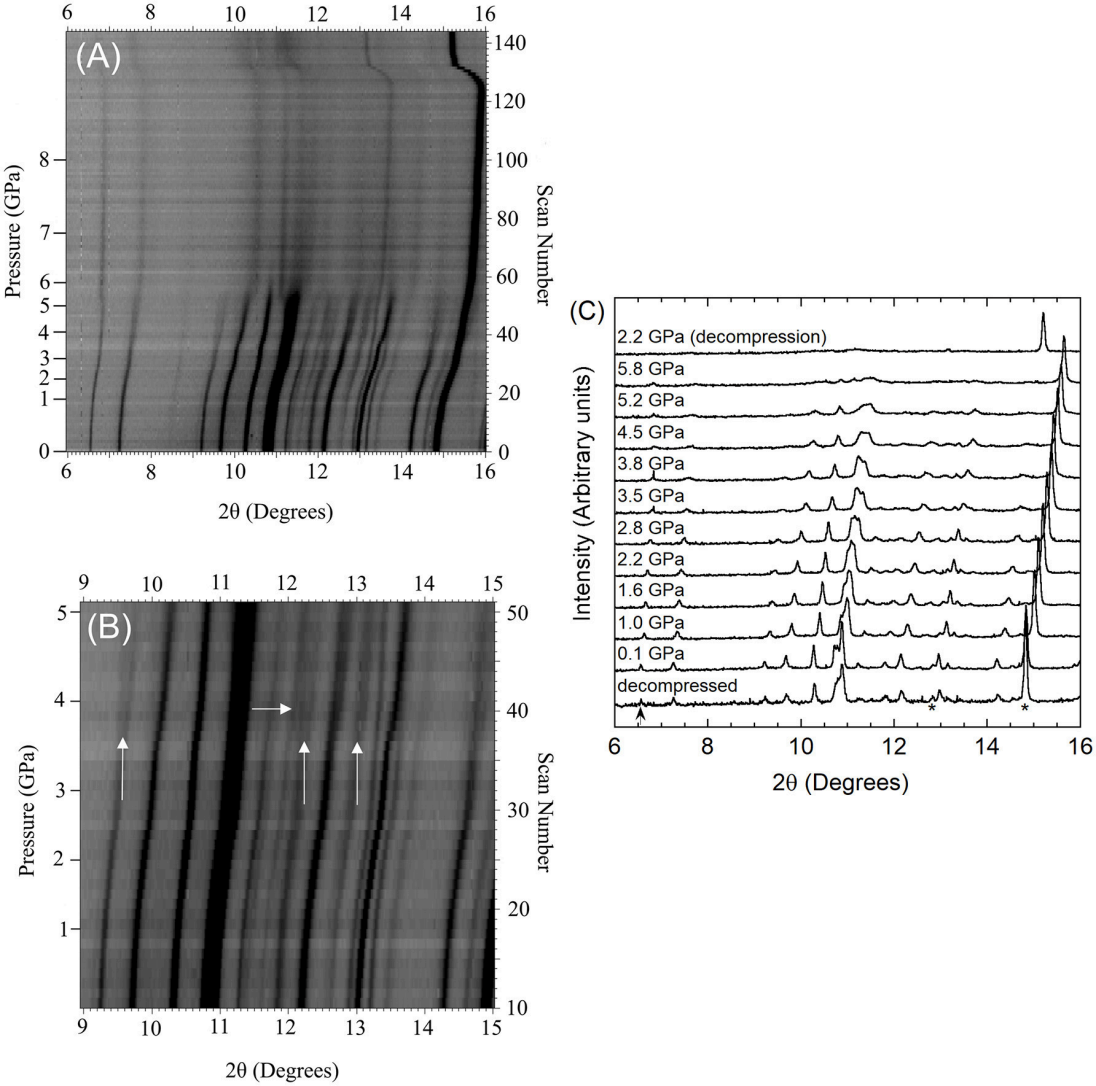
**(A,B)** Stacked 2D overlays of all high pressure data and **(C)** selected patterns for Cr_2_Mo_3_O_12_ collected during compression unless stated otherwise. In **(C)**, NaCl peaks are indicated by * in the pattern collected after decompression, and the strongest peak of the unidentified impurity phase is marked with an arrow. Vertical arrows in **(B)** mark peaks that broaden significantly compared to other Cr_2_Mo_3_O_12_ peaks, while the horizontal arrow indicates an area of peak coalescence.

**Figure 3:**
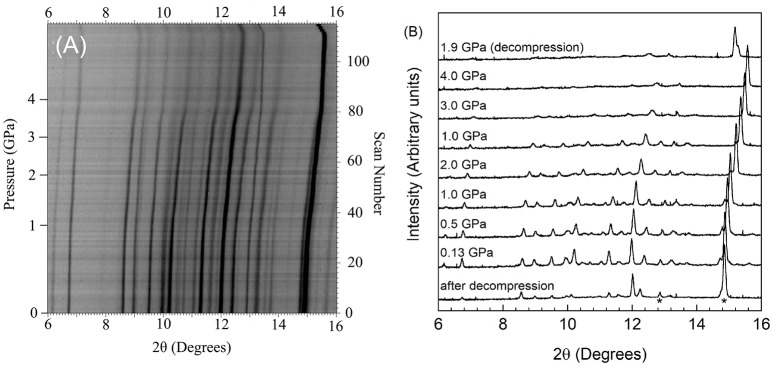
**(A)** Stacked 2D overlay of all high pressure data and **(B)** selected patterns for Pbcn-Y_2_Mo_3_O_12_ collected during compression and after decompression. In **(B)**, NaCl peaks are indicated by * in the pattern collected after decompression.

The high pressure behavior of Cr_2_Mo_3_O_12_ and Y_2_Mo_3_O_12_ was investigated *in situ* using synchrotron powder diffraction inside a DAC. During each experiment, the pressure was continuously increased. Different pressure programs were used during the two experiments, resulting in a pressure increase that was approximately twice as fast for Cr_2_Mo_3_O_12_ compared to Y_2_Mo_3_O_12_. Stack plots of all high pressure datasets and selected 1D diffraction patterns are displayed in Figures 2, 3.

The scans collected upon sealing the DAC showed that the materials were under a small amount of pressure at the beginning of data collection, as the diamond anvil cell must be tightened enough to avoid evaporation of the PTF. The pressure of the first dataset was 0.05 GPa for Cr_2_Mo_3_O_12_ and 0.13 GPa for Pbcn-Y_2_Mo_3_O_12_, respectively. Data were collected up to 8.9 GPa for Cr_2_Mo_3_O_12_ with 0.05 to 0.2 GPa increments, and up to 4.9 GPa for Pbcn-Y_2_Mo_3_O_12_ with 0.02 to 0.1 GPa increments between patterns. Inspection of the PXRD overlays showed that the peaks steadily shifted to higher angles with increasing pressure (Figures 2, 3). Visual inspection of the data did not show any obvious signs of reconstructive phase transitions to distinct crystalline high pressure polymorphs, such as peak splitting, coalescence, or abrupt changes in the pattern. At sufficiently high pressures, the peaks became progressively broader in both materials.

Diffraction data for chromium molybdate were collected up to 8.9 GPa (scan 126, Figure 2A), although the conditions became non-hydrostatic above 4.2 GPa (scan 44) due to the hydrostatic limit of isopropanol. The patterns collected at lower pressures showed comparable shifts to higher angles for all peaks, suggesting relatively isotropic compressibility of Cr_2_Mo_3_O_12_ along all three unit cell axes. Close inspection of the stacked data overlay revealed some subtle changes in the 3 to 4 GPa pressure range (see arrows in Figure 2B). Scan 38 (3.6 GPa) shows noticeable broadening of several peaks (e.g., 9.5, 12.3, and 13.2°), while other peaks coalesce with neighboring peaks (e.g., 12–12.2°). As these changes were not observed at lower pressures, this suggests that a subtle phase transition to a closely related structure occurred.

The data range displayed in Figure 2A contains two peaks that belong to the pressure standard NaCl, which are found at 12.8 and 14.8° 2θ at the beginning of the experiment (scan 0) and persist as well-defined peaks in all scans. In contrast, the Cr_2_Mo_3_O_12_ peaks start to become progressively diffuse above 5.5 GPa (scan 54), indicating the onset of disorder or amorphization. Many NTE materials have been reported to amorphize under pressure (Huang, 1998; Perottoni and da Jornada, 1998; Garg et al., 2001, 2005b; Liu et al., 2001, 2002; Secco et al., 2001, 2002a,b; Arora et al., 2004, 2005; Karmakar et al., 2004; Mukherjee et al., 2004; Paraguassu et al., 2004; Varga et al., 2005b; Gates et al., 2006; Keen et al., 2007; Catafesta et al., 2008; Cetinkol et al., 2009; Baiz et al., 2012; Torres Dias et al., 2013; Salke et al., 2018), especially under non-hydrostatic conditions, as their open frameworks allow for volume-reducing rotations of the constituent polyhedra. Under non-hydrostatic pressure, such random reorientations can become “frozen in” at relatively low pressures, resulting in progressive loss of long range order. In most materials, amorphization is irreversible upon decompression. In this work, data were also collected during decompression (scans 127-144). The final dataset during the Cr_2_Mo_3_O_12_ high pressure experiment was collected at a residual pressure of 2.2 GPa, and showed only NaCl peaks and very broad features resembling an amorphous material (Figure 2C). However, complete release of the pressure by opening the cell resulted in recovery of crystallinity, suggesting that the material does not completely disorder (Figure 2C).

Lattice constants as a function of pressure were extracted by Rietveld refinement. The pattern collected after closing the cell gave a good match to the PDF view cards of monoclinic Cr_2_Mo_3_O_12_ (01-078-1654) and cubic NaCl (01-077-2064), and these phases were used as starting models for refinements. The patterns contained intensity spikes due to sample graininess, indicating that data quality was not optimal. Throughout the data set, several unidentified peaks at 6.6, 16.9, 19.4, 25.2, and 29.5° persisted. These peaks were of low intensity (see arrow in Figure 2C for most intense peak) and could not be identified as belonging to any known impurity phases, nor any known parts of the instrumental setup such as the diamond, mounting clay, or steel. It was evident that these features shifted significantly less than the chromium molybdate peaks, and persisted as sharper peaks even above the hydrostatic limit. This behavior suggests that these peaks are either due to diffraction from a material not experiencing the same pressure as the sample, or from a very hard impurity phase. Although they could not be identified, the peaks were of such low intensity that they did not interfere with Rietveld analysis.

**Figure 4:**
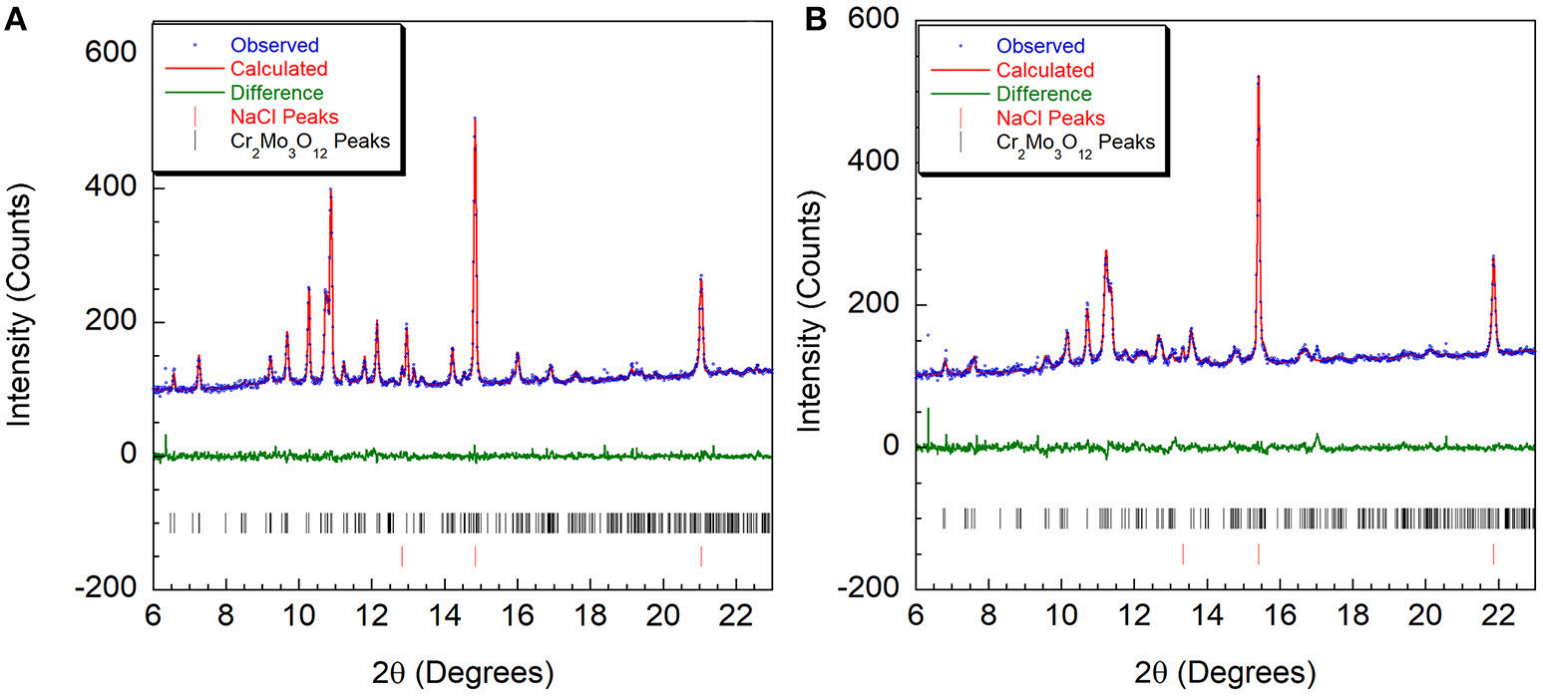
Rietveld plots of Cr_2_Mo_3_O_12_ data collected at **(A)** 0.05 GPa and **(B)** 3.7 GPa refined using the P2_1_/a structure.

**Figure 5:**
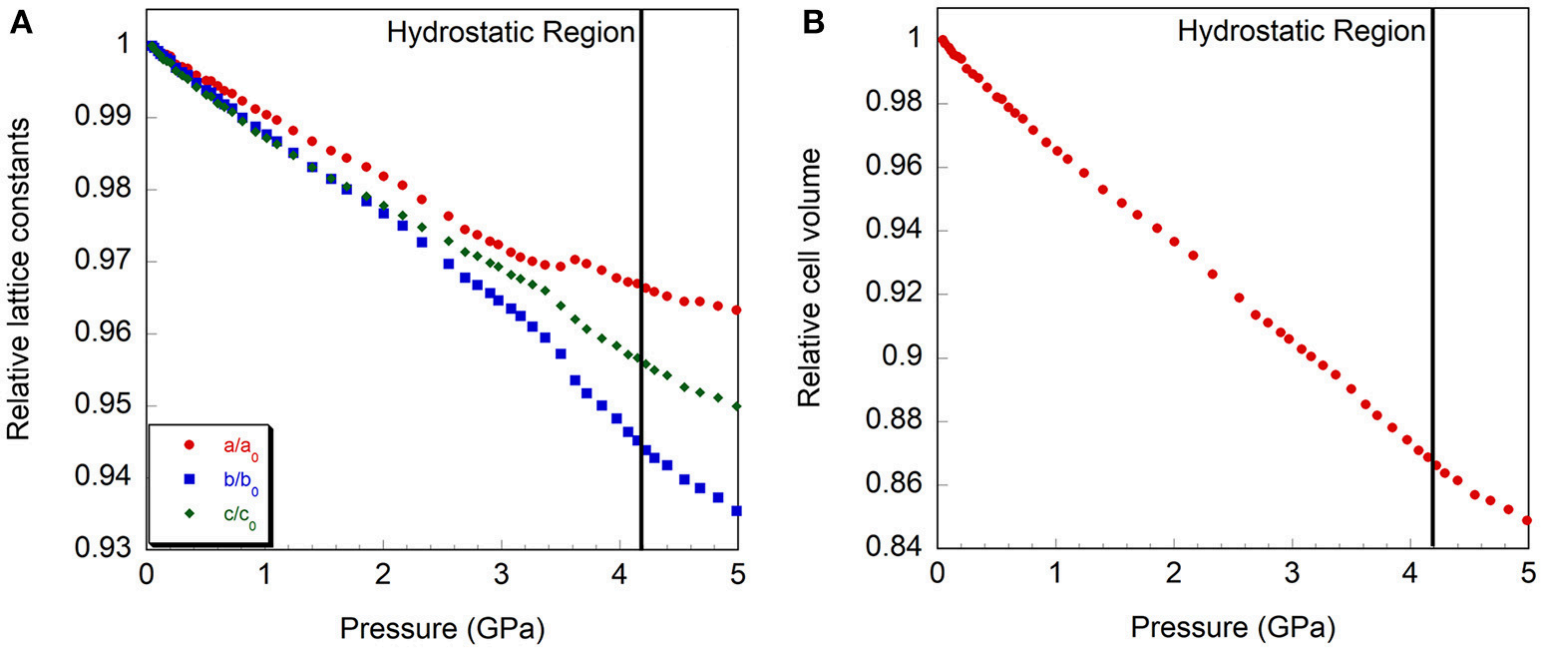
Relative compressibility of Cr_2_Mo_3_O_12_, **(A)** cell axes and **(B)** volume. The hydrostatic limit is indicated by a vertical line.

At the lowest pressure, the data were refined using the monoclinic α-Cr_2_Mo_3_O_12_ phase (Figure 4A). Bond distance restraints were necessary to vary atomic positions without losing polyhedral connectivity. Refinements with restraints resulted in slightly distorted polyhedra, but preserved the overall connectivity. After initial optimization, atom positions were fixed before running consecutive refinements to avoid potentially disastrous changes. All scans with discernible peaks could be fitted using the α-phase model. Refinement quality decreased at higher pressures as peaks began to broaden above 4.0 GPa, with only very broad features remaining above 5.0 GPa (Figure 2C). This pressure range extends above the hydrostatic limit of isopropanol, which is 4.2 GPa. The changes in lattice parameters and volume (Figure 5) were extracted up to 5.0 GPa. Linear behavior was observed up to ~3.5 GPa, at which point a discontinuity or a change in slope occurred for all lattice parameters. A small but distinct increase in the a parameter was observed, followed by a continued decrease at a slightly lower rate. In contrast, a small stepwise decrease of the b-parameter preceded a steepening of the compressibility slope. The evolution of the c-parameter was almost continuous. This suggested a subtle structural distortion, which is supported by the observed anisotropic peak shifts in the data (Figure 2B). A full structural refinement of a selected dataset was carried out above this subtle phase transition using the ambient monoclinic structure of Cr_2_Mo_3_O_12_ as a starting point. Only the metal positions could be varied despite using bond distance restraints due to the lower data quality. A good fit was achieved (Figure 4B), corroborating that a closely related structure was formed. Similar transitions have been observed in Al_2_Mo_3_O_12_, AlGaMo_3_O_12_, Fe_2_Mo_3_O_12_, and FeAlMo_3_O_12_ (Young et al., 2016), where the compressibility of the unit cell axes changed at pressures between 1.5 and 3 GPa, but structural refinements using the original α-phase model remained stable. In addition, these four compounds underwent a second transition to a γ-phase at pressures between 2.7 and 4.9 GPa, which involved a small discontinuity in several lattice parameters as well as the cell volume. All three phases could be described with the ambient pressure P2_1_/a monoclinic structure model. The pressure for the α- to α'- and α'- to γ-transitions correlated well with the average ionic radius of the A^3+^ cation in the previous study, with larger cations resulting in a lower transition pressure. The α- to α'- transition was in all cases accompanied by significant stiffening of the c-axis, while the a- and b axes softened, stiffened or remained largely unaffected depending on composition. In contrast, c-axis compressibility remained similar in the α'- and γ-phases, while the b-axis softened by about 30%.

The small discontinuity observed in the lattice constants of Cr_2_Mo_3_O_12_ suggests that the transition observed should be compared to the formation of the γ-phase in Al_2_Mo_3_O_12_, AlGaMo_3_O_12_, Fe_2_Mo_3_O_12_ and FeAlMo_3_O_12_, even though no stepwise decrease in cell volume is observed due to the combination of significant a-axis stiffening, b-axis softening and unaffected c-axis compressibility. The change in compressibility of the b- and c-axes between α-Cr_2_Mo_3_O_12_ and γ-Cr_2_Mo_3_O_12_ is comparable to what was observed in the previously studied compositions, while the significant stiffening of the a-axis is much more pronounced than the changes in Al_2_Mo_3_O_12_, AlGaMo_3_O_12_, Fe_2_Mo_3_O_12_, and FeAlMo_3_O_12_. The transition pressure of ~3.5 GPa is similar to the 3.2 GPa transition pressure reported for the formation of γ-FeAlMo_3_O_12_ (Young et al., 2016). The ionic radius of Cr^3+^ in octahedral coordination is 61 pm, which is comparable to the average ionic radius of an equimolar mixture of Fe^3+^ and Al^3+^ in octahedral coordination (59.5 pm).

Separate compressibility constants were extracted for the pressure ranges corresponding to the α- and γ-phases of Cr_2_Mo_3_O_12_ to avoid contributions from the phase transition. Compressibility constants for α-Cr_2_Mo_3_O_12_ were extracted from 0.05 to 3.4 GPa, and were found to be β_α, *a*_ = 0.93 ± 0.01 × 10^−2^ GPa^−1^, β_α, *b*_ = 1.19 ± 0.01 × 10^−2^ GPa^−1^, β_α, *c*_ = 1.01 ± 0.01 × 10^−2^ GPa^−1^, and β_α, *v*_ = 3.13 ± 0.02 × 10^−2^ GPa^−1^. For γ−*Cr*_2_*Mo*_3_*O*_12_, these values changed to β_γ, *a*_ = 0.64 ± 0.02 × 10^−2^ GPa^−1^, β_γ, *b*_ = 1.51 ± 0.04 × 10^−2^ GPa^−1^, β_γ, *c*_ = 0.99 ± 0.02 × 10^−2^ GPa^−1^, and β_γ, *v*_ = 3.08 ± 0.04 × 10^−2^ GPa^−1^ for the 3.6 to 4.5 GPa pressure range, indicating that the overall volume compressibility decreased slightly. Above 4.5 GPa, stiffening of all lattice constants was observed. The compressibility of α-Cr_2_Mo_3_O_12_ is close to isotropic, with b slightly softer than the other axes. This is similar to other scandium tungstate materials (Liu et al., 2003; Varga et al., 2006; Young et al., 2016). Above the subtle phase transition, the compressibility becomes considerably more anisotropic. The bulk moduli of the α- and γ-phases were found to be 28.7 ± 0.4 GPa and 26.3 ± 1.2 GPa, respectively, indicating almost negligible stiffening at high pressure.

Yttrium molybdate patterns were collected at pressures up to 4.9 GPa (scan 110), which is slightly higher than the hydrostatic limit of isopropanol. No distinct phase transitions were observed in the scan overlay (Figure 3A), and all peaks shifted uniformly to higher angles with increasing pressure, indicating isotropic compressibility of all lattice constants. Peaks belonging to the NaCl pressure standard remained sharp throughout the experiment, while the Y_2_Mo_3_O_12_ peaks became broader and less intense above 3.8 GPa (scan 80), which is close to the hydrostatic limit for isopropanol. Peaks remained broad throughout decompression (scans 111-115) to 1.9 GPa. Integrated patterns showed broader and weaker features as pressure was increased, and the scan collected at the end of the high pressure run [1.9 GPa (decompression)] remained largely featureless except for NaCl peaks (Figure 3B). Upon complete release of pressure by opening the cell, crystallinity was recovered, but peaks remained broader and weaker, indicating that some irreversible structural damage occurred.

**Figure 6:**
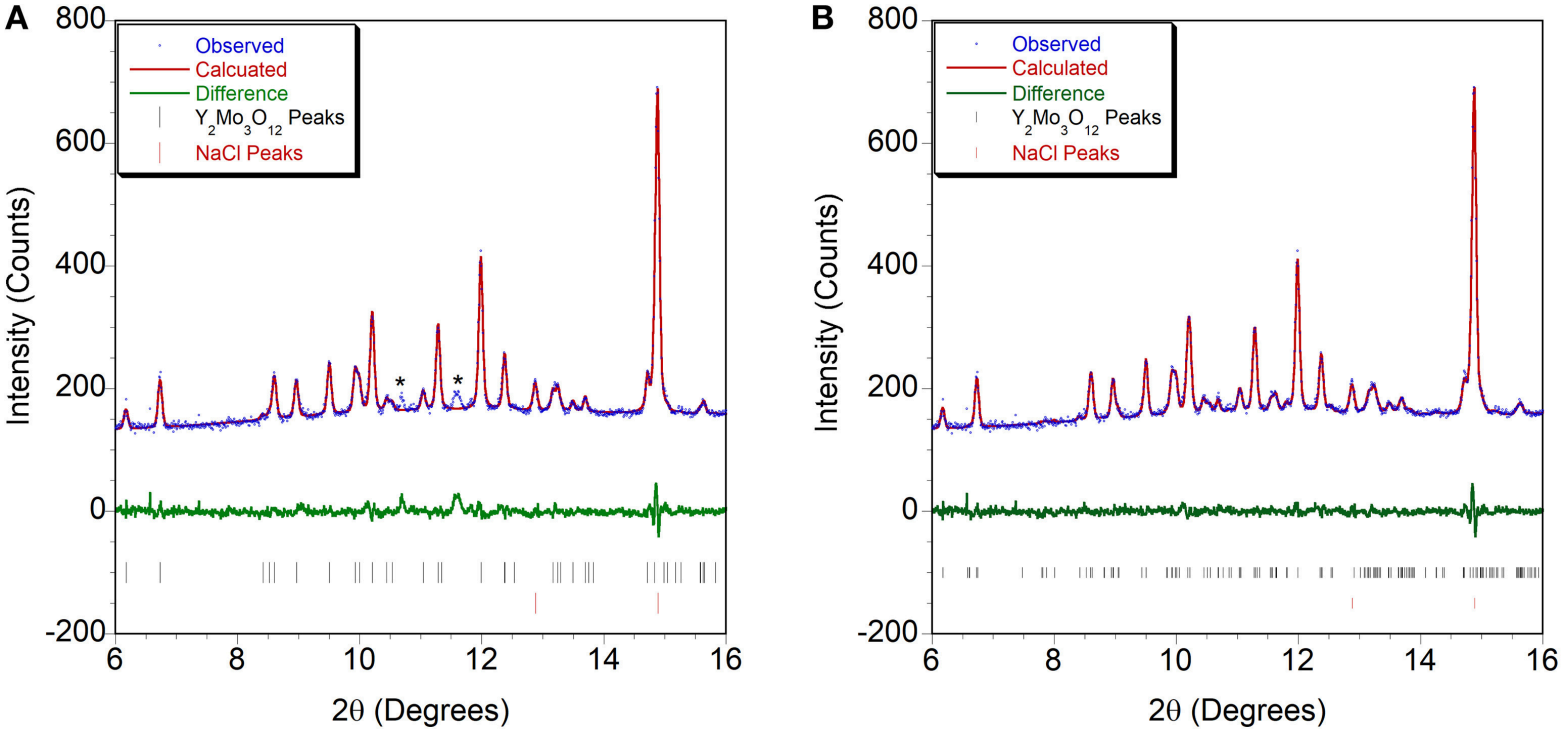
Rietveld plots of PXRD data collected at 0.13 GPa refined using structural models in space groups **(A)** Pbcn and **(B)** P2_1_/a. * indicates peaks that cannot be accounted for by the orthorhombic cell.

**Figure 7:**
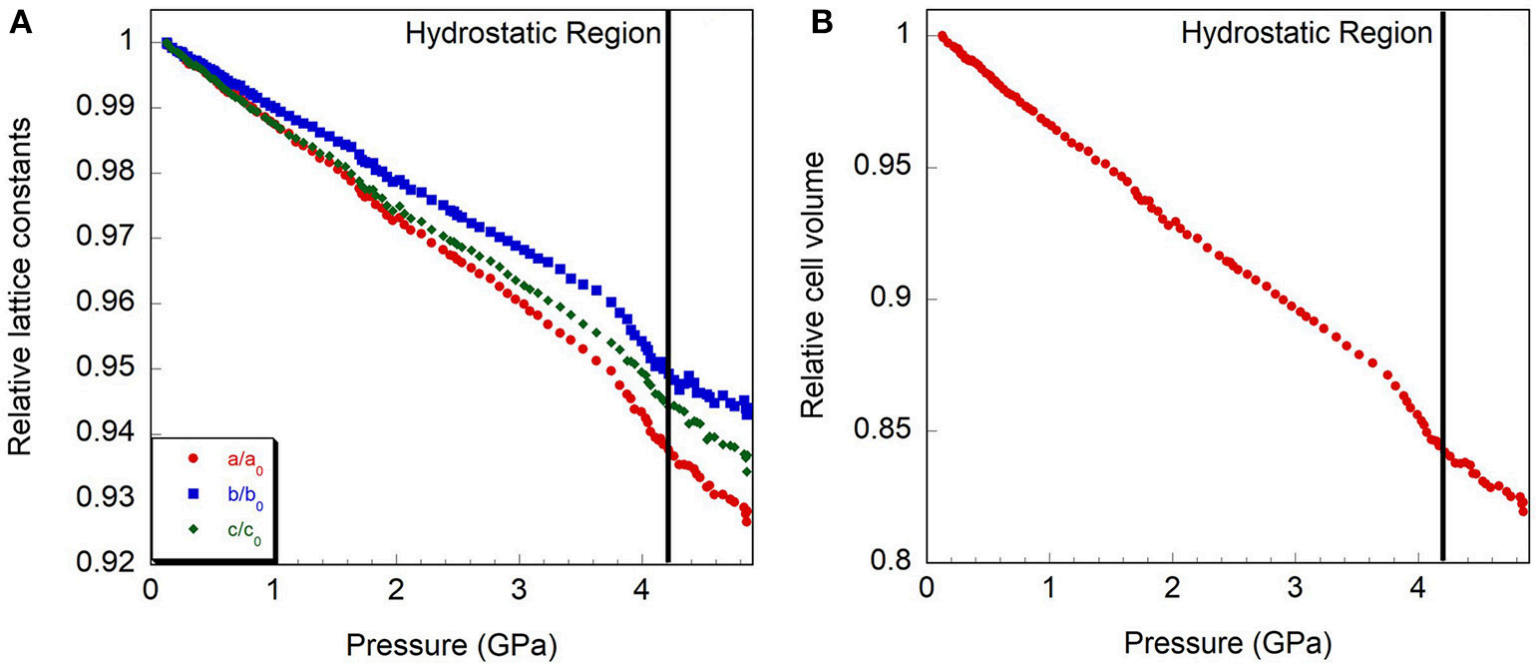
Relative compressibility of monoclinic Y_2_Mo_3_O_12_, **(A)** cell axes and **(B)** volume. The hydrostatic limit is indicated by a vertical line.

Initially, a Rietveld refinement starting from the Pbcn structure was attempted for the as-closed dataset collected at 0.13 GPa. A reasonable fit could only be achieved after varying the atomic positions. This resulted in a cell with lattice constants of a = 13.53 Å, b = 9.81 Å, and c = 9.94 Å. However, two peaks remained unaccounted for (Figure 6A), and inspection of the refined structure revealed that the atoms had moved far enough to no longer form recognizable polyhedra. This held true even when constraints were applied. Additionally, the unit cell parameters indicated that a 4.4% reduction in unit cell volume had occurred when compared to the ambient pressure cell constants. This reduction in unit cell volume was much higher than expected based on the compressibility of other orthorhombic A_2_Mo_3_O_12_ compounds, and suggested that a transition to the structurally related higher density P2_1_/a polymorph may have occurred below 0.13 GPa. This agrees with a previous Raman study by Torres Dias et al. (2013), which suggested that Pbcn-Y_2_Mo_3_O_12_ underwent a transition to a lower symmetry phase below 0.3 GPa. It is not surprising that this transition would occur at such a low pressure, as previous studies have shown that Sc_2_W_3_O_12_ (Garg et al., 2005a; Varga et al., 2005b, 2006; Cetinkol et al., 2008), Sc_2_Mo_3_O_12_ (Varga et al., 2005a) and Al_2_W_3_O_12_ (Varga et al., 2005a) behave similarly and undergo this transition at 0.3 GPa, 0.25 GPa and 0.1 GPa, respectively. Initial unit cell parameters for the corresponding monoclinic unit cell were estimated based on the known transformation matrix (Evans and Mary, 2000), and combined with the atomic coordinates of α−Fe_2_Mo_3_O_12_ as a starting model for a Rietveld refinement. Soft distance restraints were applied to stabilize the refinement, which resulted in an excellent fit with final unit cell parameters of a = 16.726 Å, b = 9.943 Å, c = 19.643 Å, and β = 125.77°. Reasonable bond distances that preserved the polyhedral connectivity were obtained, confirming that a phase transition to monoclinic ε-Y_2_Mo_3_O_12_^1^ had occurred (Figure 6B). Some distortion of the polyhedra was evident, although this could also be a result of the limited data quality. The final atomic coordinates obtained for the 0.13 GPa dataset are provided in Table 2. As no evidence of further phase transitions was observed, consecutive Rietveld refinements of all scans up to the highest pressure were carried out using the monoclinic ε-Y_2_Mo_3_O_12_ cell. Atomic positions were fixed for these consecutive refinements. Linear compressibility was observed up to 3.7 GPa (Figure 7). Above this pressure, peak broadening and the resulting deteriorating refinement quality made extraction of lattice constants unreliable, which was evident from significant increases in their statistical errors. While quantitative analysis at pressures above 3.7 GPa is not feasible, the evolution of lattice parameters suggests that the material softens in this pressure range. Most materials get stiffer at high pressures due to reduction of empty space upon compression. However, pressure induced softening has been observed in a number of NTE material (Pantea et al., 2006; Fang and Dove, 2013, 2014; Fang et al., 2013; Morelock et al., 2013; Gallington et al., 2014; Hancock et al., 2015; Alabarse et al., 2017; Hester et al., 2017b; Ticknor et al., 2018), and has been linked to the facile polyhedral rotations that give rise to their expansion behavior. In many cases, amorphization is preceded by pressure induced softening as well. To avoid contributions from this region, the relative compressibilities were extracted for the 0.13 to 3.7 GPa pressure range, giving values of β_ε, *a*_ = 1.39 ± 0.004 × 10^−2^ GPa^−1^, β_ε, *b*_ = 1.09 ± 0.003 × 10^−2^ GPa^−1^, β_ε, *c*_ = 1.26 ± 0.01 × 10^−2^ GPa^−1^, and β_ε, *v*_ = 3.57 ± 0.01 × 10^−2^ GPa^−1^. In contrast to other materials in the scandium tungstate family that are most compressible along the b-axis, the P2_1_/a-Y_2_Mo_3_O_12_ phase is stiffest along the b-axis. The bulk modulus was estimated to be 24.8 ± 0.2 GPa, which is similar to other A_2_M_3_O_12_ compositions studied.

**Table 2:** Atomic coordinates for monoclinic Y_2_Mo_3_O_12_ at 0.13 GPa (space group P2_1_/a).

**Name**	**X**	**Y**	**Z**
Y1	0.37 (5)	0.96 (5)	0.32 (2)
Y2	0.38 (5)	0.46 (5)	0.05 (3)
Y3	0.13 (3)	0.48 (5)	0.18 (2)
Y4	0.11 (5)	0.97 (5)	0.43 (4)
Mo1	−0.01 (3)	0.25 (4)	0.49 (3)
Mo2	0.37 (4)	0.13 (3)	0.14 (2)
Mo3	0.13 (3)	0.14 (5)	0.26 (2)
Mo4	0.14 (3)	0.61 (4)	0.37 (2)
Mo5	0.35 (3)	0.64 (4)	0.20 (2)
Mo6	−0.02 (3)	0.72 (5)	0.01 (2)
O1	0.58 (19)	0.40 (20)	0.00 (11)
O2	0.00 (20)	0.43 (18)	0.16 (9)
O3	0.90 (13)	0.20 (20)	0.10 (12)
O4	0.74 (18)	0.43 (13)	0.06 (12)
O5	0.52 (16)	0.37 (17)	0.14 (12)
O6	0.70 (20)	0.46 (16)	0.29 (10)
O7	0.46 (14)	0.12 (17)	0.40 (13)
O8	0.18 (15)	0.30 (20)	0.26 (11)
O9	0.57 (16)	0.40 (20)	0.47 (10)
O10	0.42 (12)	0.30 (20)	0.98 (13)
O11	0.12 (11)	0.38 (17)	0.08 (12)
O12	0.40 (20)	0.38 (16)	0.49 (11)
O13	0.87 (14)	0.39 (16)	0.23 (13)
O14	0.25 (17)	0.01 (19)	0.54 (12)
O15	0.15 (13)	0.04 (16)	0.35 (12)
O16	0.50 (20)	0.95 (17)	0.34 (10)
O17	0.70 (20)	0.97 (19)	0.16 (12)
O18	0.65 (15)	0.89 (14)	0.28 (9)
O19	0.97 (12)	0.93 (14)	0.30 (9)
O20	0.04 (12)	0.32 (18)	0.60 (10)
O21	0.11 (14)	0.80 (20)	0.36 (12)
O22	0.06 (17)	0.66 (19)	0.13 (13)
O23	0.37 (15)	0.61 (17)	0.12 (9)
O24	0.31 (12)	0.80 (20)	0.22 (9)

The fact that the monoclinic phases of Cr_2_Mo_3_O_12_ and Y_2_Mo_3_O_12_ remain stable up to high pressures has interesting implications for potential uses of these materials in controlled thermal expansion applications. While the monoclinic phases observed throughout the high pressure experiment display positive expansion under ambient pressure, it is known that heating can induce the monoclinic-to-orthorhombic transition in almost all monoclinic A_2_M_3_O_12_ compositions. This suggests that applications at increased temperatures may see a reversion to the orthorhombic structure under moderate pressures. Such temperature/pressure dependent phase transitions were observed for ZrV_2_O_7_ (Gallington et al., 2017), which shows positive expansion in a 3 × 3 × 3 superstructure below 100 °C and NTE in a simple cubic cell at higher temperatures. Chapman and Chupas have estimated that NTE materials are likely to be subjected to pressures of ~1 GPa when used in composites (Chapman and Chupas, 2007). At this pressure, the majority of A_2_M_3_O_12_ compositions studied adopt the monoclinic P2_1_/a phase (Table 1). However, heating under pressure can also provide the necessary energy to overcome kinetic barriers to the formation of denser polymorphs, the existence of which has been reported for many compositions (Table 1). Such transformations may affect potential uses of Y_2_Mo_3_O_12_, as the thermodynamically stable polymorph (Pba2) below 550 °C is denser than the Pbcn- and P2_1_/a-phases (Gates and Lind, 2007). In contrast, no denser phases are known for Cr_2_Mo_3_O_12_. It would be interesting to study the behavior of this material in pressure-temperature space to determine the orthorhombic-monoclinic phase boundary.

## Conclusions

High pressure studies of Pbcn-yttrium molybdate and chromium molybdate were conducted up to 4.9 and 8.9 GPa, respectively. Pbcn-yttrium molybdate underwent a phase transition to a monoclinic P2_1_/a phase below 0.13 GPa. This is the first time that this polymorph has been structurally characterized. The monoclinic P2_1_/a structures of both compounds are remarkably stable and do not undergo abrupt structural phase transitions upon compression. This is unexpected, as most previously investigated isostructural A_2_M_3_O_12_ materials underwent at least one distinct pressure induced phase transition. Changes in the evolution of lattice constants and compressibility as a function of pressure suggest that Cr_2_Mo_2_O_12_ may undergo a subtle structural distortion similar to what is observed for Al_2_Mo_3_O_12_, AlGaMo_3_O_12_, Fe_2_Mo_3_O_12_, and FeAlMo_3_O_12_, while no such changes were observed for P2_1_/a-Y_2_Mo_3_O_12_.

It is currently unclear what property of the A-site cation causes the remarkable stability of the P2_1_/a polymorph in these compounds and thus whether other compositions may exhibit this behavior as well. While the monoclinic phases are not expected to exhibit NTE, their stability under pressure has important implications for composite fabrication, as the facile back-conversion to the orthorhombic phase during decompression or heating would reverse any detrimental changes that could occur during composite formation. These materials could find applications in controlled thermal expansion applications at slightly elevated temperatures, which is expected to favor the orthorhombic NTE phase even under pressure. While Y_2_Mo_3_O_12_ may convert to the denser Pba2-structure at moderate temperatures and pressures, the exceptional stability of Cr_2_Mo_3_O_12_ upon compression with respect to both formation of denser crystalline polymorphs and amorphization makes this material an attractive target.

## Author contributions

CL devised the experiments, trained students on synthesis and characterization, participated in high pressure experiments, data collection and analysis as well as writing of paper. LY selected samples for high pressure experiments, participated in high pressure experiments, data collection and analysis as well as writing of paper. JG participated in high pressure experiments, data collection and analysis.

### Conflict of interest statement

The authors declare that the research was conducted in the absence of any commercial or financial relationships that could be construed as a potential conflict of interest.

## Footnotes

^1^Under ambient conditions, Y_2_Mo_3_O_12_ may form Pba2 α−Y_2_Mo_3_O_12_ or Pbcn β−Y_2_Mo_3_O_12_, depending on synthetic conditions. High pressure studies of both materials have been published. Two high pressure phases were observed to evolve from the Pba2 phase, γ and δ. The Pbcn phase was shown to undergo a transition to the monoclinic P2_1_/a structure, designated the ε-phase.
